# Two Cases in Practice

**Published:** 1862-04

**Authors:** J. B. Hoag

**Affiliations:** Windfall, Tipton Co., Ind.


### TWO CASES IN PRACTICE.

By J. B. HOAG, M. D., Windfall, Tipton Co., Ind.

About the 1st of August, 1860, whilst visiting the town where I now reside, I was requested to visit a child which had previously been under the charge of a so-called "Eclectic," who had pronounced it a case of phrenitis, and prognosticated a fatal result. Contrary to the usual habit of his class, he consented to my taking charge of the case.

In a community a large number of whom pinned their faith implicitly upon the Eclectic's sleeve, it may be imagined the noisy clamor of tongues which ensued. It may be left to surmise how the "permanent committee" of gossips reported a chorus of "chin music."

Alas for human nature!—but it seemed as though, whilst astounded at my presumption in daring to come athwart the pathway of their favorite, they almost forgot to wish that the child might by some means be spared to the widowed mother.

The patient, a little girl of three years, I found with a high fever, in a comatose condition from which it had been impossible to arouse her for forty-eight hours. From the entire absence of evidences of phrenitis, and the history of the case, there was no difficulty in diagnosing the real character of the difficulty to be bilious remittent, aggravated by the nature of the gastric and intestinal contents, evidently of a noxious character, and probably also by the presence of worms.

Much to the comfort of the mother, but, I regret to add, exceedingly to the discomfort and chagrin of our eclectic friends, I gave a favorable prognosis.

One of the ladies of the "permanent committee" immediately conveyed the budget to Dr. S., the eclectic, of course not forgetting the worms. Whereupon S. avowed his deliberate intention to eat every worm passed more than three. Three worms were, I suppose, allowed as the normal total for the three years of the patient.

Ordered the child a free cathartic dose of calomel, with a small quantity each of ipecac and gamboge. Applied rubefacients (horse radish leaves) to the nape of the neck, hands and feet.

In a few hours the medicine operated; the first passage the mother remarked, "looking like the white of an egg, only it was green as grass." As soon as this was discharged, the child roused from its stupor, and raising one of its feet, called, "Ma, ma," the first words it had spoken for more than two days.

The draughts were removed, and I then exhibited Sp. Nitri Dul., combined with Tr. Verat. Virid., and repeated the mercurial cathartics (principally Hyd. Cu. Creta) followed by Ol. Ricini. After a short time, the fever began to have short intermissions, during which I gave as large doses of Sulph. Quiniæ as I thought the child would bear. During the febrile paroxysms, continuing the mixture of Sp. Nit. Dul. and Tr. Verat. Virid.

It may be mentioned that within the first three days of treatment, the child passed a considerable number of worms, some alive and others dead—which, however, the eclectic gentleman failed to make good his promise with regard to. Convalescence took place rapidly and without any marked symptoms.

Some time subsequently, it was attacked with "congestive chills" of a dangerous type, but they were relieved by the usual treatment, and it is now in the enjoyment of excellent health.

Among the indescribably ludicrous features which, from the very outset, marked the history of this case, it may be mentioned, that the astonished Eclectic now declares that he never called it Phrenitis; that he never said it would die, and on the whole seems to doubt whether he was ever called to see it at all, or, if so, whether it was sick any how!—So goes the world.

In the same neighborhood, and under the oversight of the same Dr. S., was a case of very dissimilar character, but which to me shows the efficiency of active treatment for real disease. Many cases tend spontaneously to health, and these make the fortunes of Homœopaths and quacks of other shades; but some cases demand positive treatment, as I think the following illustrates.

J. G., unmarried, had been troubled for several years with dysmenorrhœa. I found her anæmic, variable and capricious appetite, feeble pulse, cold extremities, habitual constipation, evidently dependent on hepatic torpor. Menstrual discharges scanty, painful and irregular.

Ordered a free cathartic dose of Cal., Aloes, Ipecac and Capsic., to be followed by Ol. Ricini. After thus unloading the canal and correcting the secretions, I prescribed the following mixture, ℞ Tr. Guaiaci ℥ij; Tr. Mur. Ferri ℥j; Tr. Myrrhæ ℥j; Tr. Aloes ℥iij; Tr. Sanguinariæ ℥iss; M. a teaspoonful three times a day.

Also, ℞—Carb. Ferri, part. ij ; Carb. Soda, Pulv. Zingib. and part. j. M. S.—As much as would lie on a three cent piece, half an hour after each meal. Hot foot baths every night—a heaping table spoonful of mustard to a sufficient quantity of hot water. On the approach of the period, to drink freely of a hot decoction of wild ginger and tansy, and to apply a fomentation of the latter over the uterine region.

On the access of the first subsequent period, the discharge was more free, but still accompanied with considerable pain, which was soon mitigated by full doses of Pulv. Doveri and Camphor.

No modification of the treatment became necessary, as she speedily became perfectly restored. Is now married and enjoys better health than she had before for years.

The origin of the trouble illustrates a peculiarity of feminine tendencies. It appears that some three years previous, a church dedication was to occur at eight miles distant, which the young people of the neighborhood were anxious to attend The untimely advent of the menstrual period made the young lady fearful that she should be obliged to remain behind. The night previous to the meeting she stood for nearly an hour in a tub of ice water; the result, naturally, being immediate cessation of the flow and ruined health for years.

The only wonder is, that even more serious results did not ensue. But who can account for women ?

